# Supplementary material for: High-throughput characterization of bacterial responses to complex mixtures of chemical pollutants
Source: Nat Microbiol. 2024 Mar 18;9(4):938–48. doi: 10.1038/s41564-024-01626-9 (PMC10994839; doi:10.1038/s41564-024-01626-9)
Supplement: Supplementary file 1 — Supplementary Figs. 1–9, Supplementary Tables 1–4 and Supplementary Notes. [file 41564_2024_1626_MOESM1_ESM.pdf]

nature microbiology

Article

https://doi.org/10.1038/s41564-024-01626-9

High-throughput characterization of bacterial responses to complex mixtures of chemical pollutants

In the format provided by the authors and unedited

Supplementary information for: High throughput characterization of bacterial responses to complex mixtures of chemical pollutants

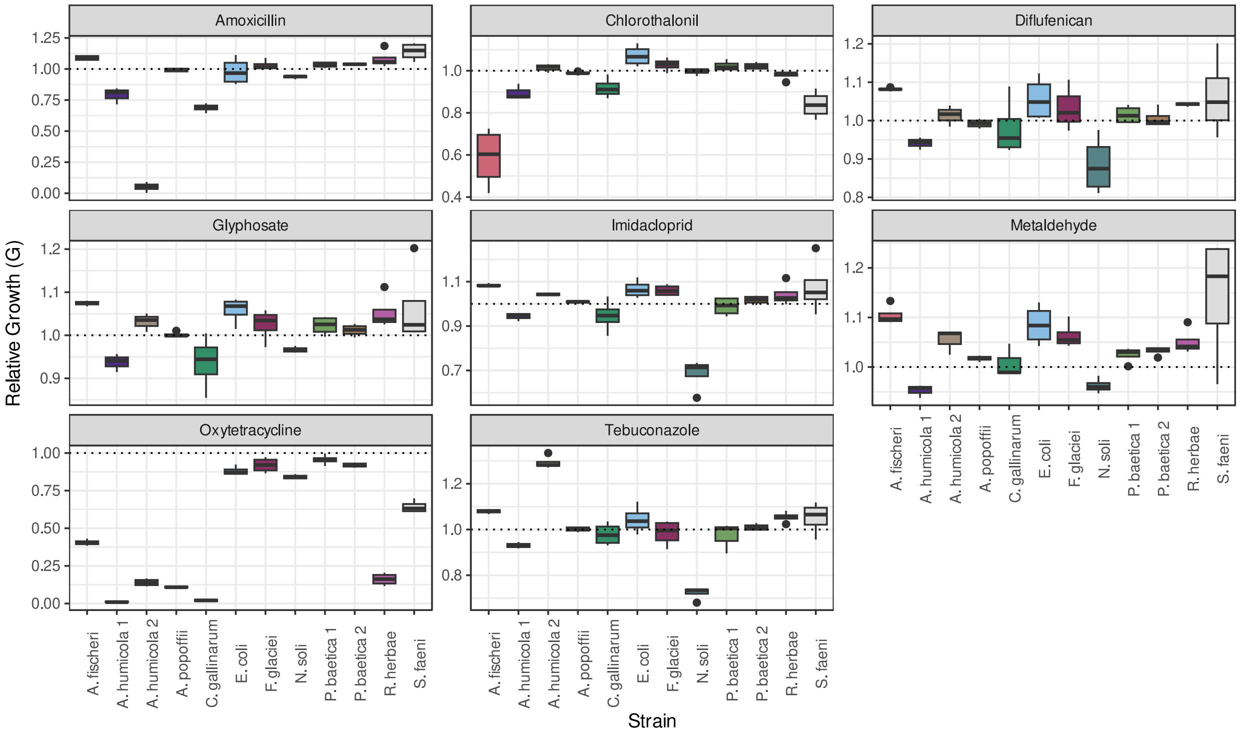

Figure S1 Responses of bacteria to single stressors. *Boxplots of replicate measurements of growth for each strain, in the presence of each stressor (n=4 biologically independent experiments). Central line marks the median, box corresponds to the first and third quartiles (the 25th and 75th percentiles), whiskers extend to the largest and smallest values no further than 1.5× the interquartile range (IQR). Datapoints beyond the whiskers are plotted individually. Growth expressed as AUC relative to a mean of control measurements for that strain (G). Dashed line marks 1, i.e., no effect on growth compared to control. Each chemical stressor has some effect on growth in at least one strain of bacteria used at the experimental dosage (0.1mg/L).*

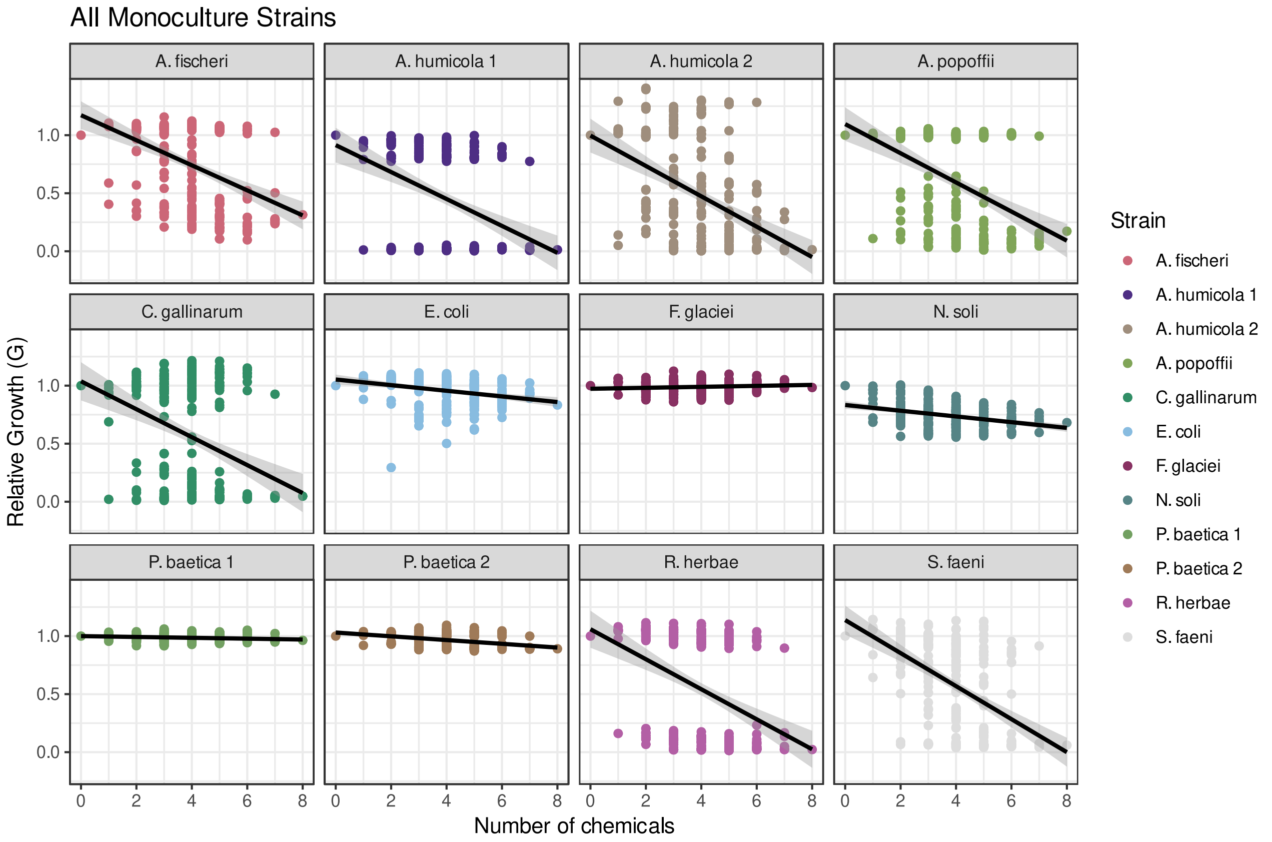

Figure S2 Variation in the response of different bacteria to chemical mixtures of increasing complexity. *Here we show the responses of each bacterial strain in monoculture to every mixture of chemicals, given as growth relative to control growth (points are the mean of 4 biologically independent experiments). Some strains show the bimodality of responses (e.g. R. herbae, C. gallinarum), others show little response to the chemicals at all (e.g. P. baetica 1 & 2). Black line shows a linear model prediction, grey band is the 95% confidence level interval for the linear model prediction.*

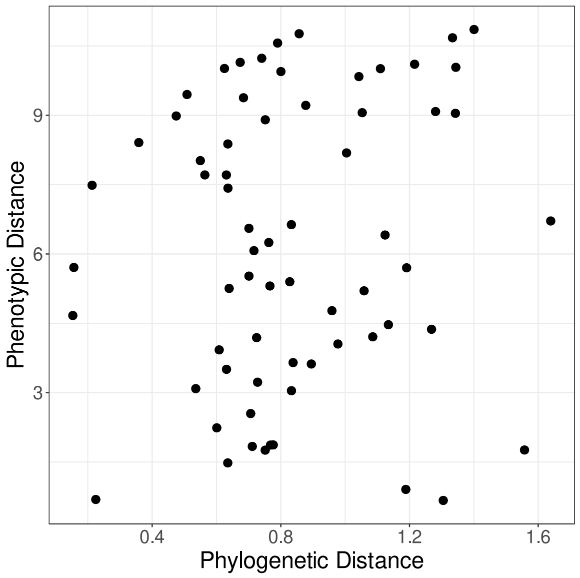

Figure S3 Mantel test distances. *Pair-wise phenotypic (responses to all chemical mixtures) distance plotted against pair-wise phylogenetic distance. We find no significant correlation between the phylogenetic and phenotypic distance matrices (One-sided Mantel test, p = 0.154).*

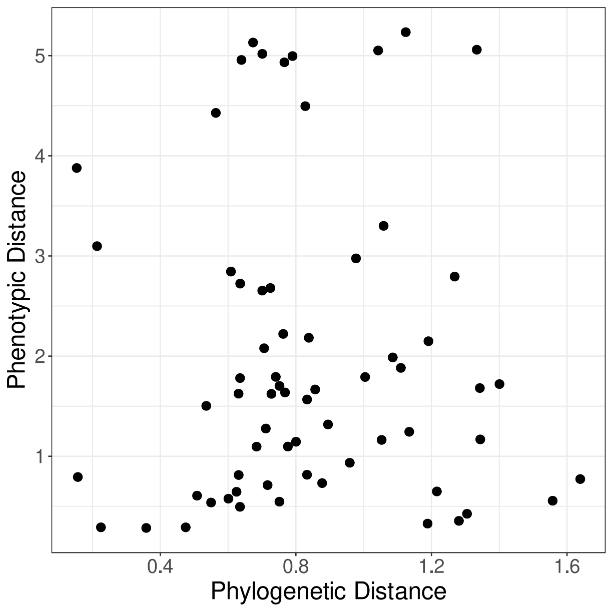

Figure S4 Mantel test distances without oxytetracycline. *Pair-wise phenotypic distance calculated after excluding the chemical mixtures containing oxytetracycline, plotted against pair-wise phylogenetic distance. We find no significant correlation between the phylogenetic and phenotypic distance matrices (One-sided Mantel test, p = 0.322).*

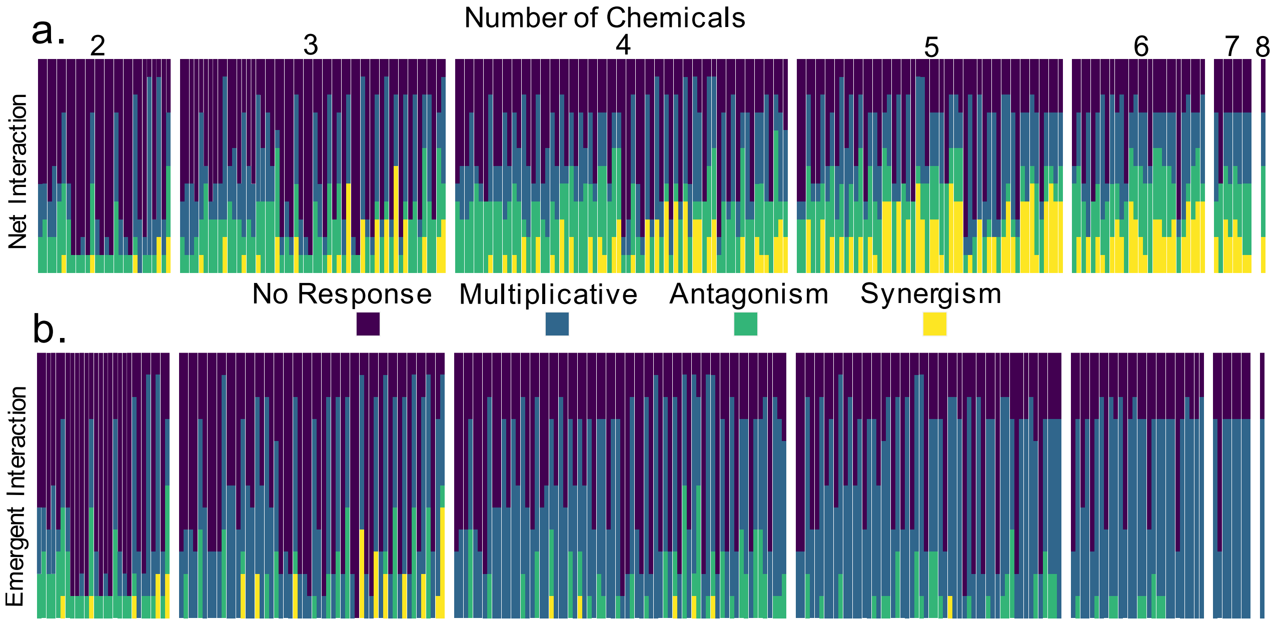

Figure S5 Interactions vary between strains. *Bars count the number of occurrences of each interaction type across the 12 strains tested for each chemical stressor combination, grouped by the number of stressors in the mixture. Each stacked bar represents one of the 247 chemical mixtures in our experiment (i.e., all treatments with more than one stressor). Colours show whether there was no response to the mixture (purple), whether the response was multiplicative (blue, a significant effect on growth, but no interaction according to our multiplicative null model) or whether an antagonistic (green) or synergistic (yellow) interaction was found. Net interactions (**a.**) are more prevalent in more complex mixtures, but are not consistent across strains (the same chemical mixture elicits qualitatively different interactive effects on different strains). Emergent interactions (**b.**) are less prevalent in more complex mixtures, but are more consistent among strains (where a higher order emergent interaction occurs, multiple strains often show the same interaction). Two-way interactions are identical for both net and emergent effects, as the null model is the same in both cases.*

Prevalence of interactive effects

Despite the apparent lack of generalisability of interactions, there were certain mixtures of chemicals which produced interactions across a number of the strains tested here. The chemical pairing resulting in the most interactions observed was oxytetracycline (antibiotic) and tebuconazole (fungicide), henceforth "O-T". However, the direction of interactions were not consistent, with four strains showing an antagonism and two strains showing a synergism for this pairing (Figure S6). Regardless of the direction of interaction, where a two-way interaction in mixtures of these chemicals occurred, the same interaction tended to persist up into higher complexity mixtures, following similar patterns for different species (Figure S6). All four species showing an antagonistic O-T interaction also showed an emergent synergism when metaldehyde was added to the mixture (Figure S7). Here, the addition of metaldehyde, while showing no direct interactions with oxytetracycline or tebuconazole in pair, produces a response which opposes the O-T pairwise interaction. Moreover, there were also 4-way antagonistic interactions observed in each of these species in mixtures which included metaldehyde and oxytetracycline (but excluded tebuconazole; Figure S7).

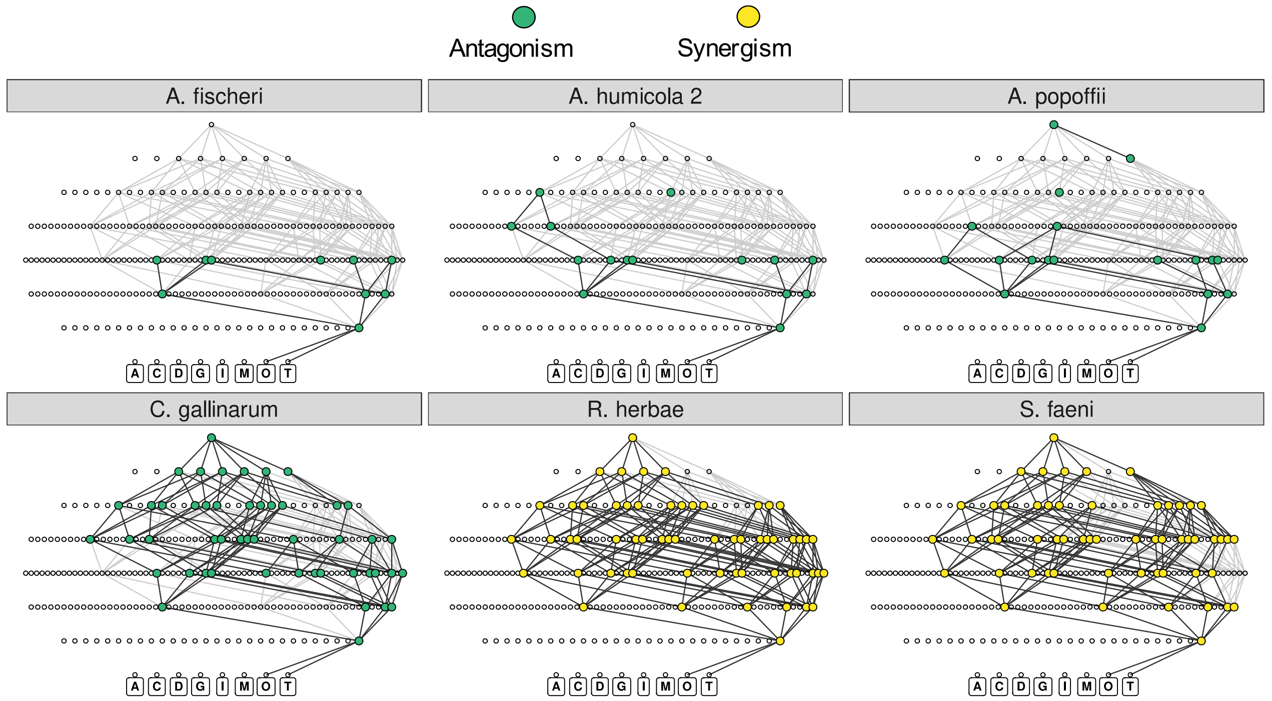

Figure S6 Oxytetracycline (O) and Tebuconazole (T) net interactions persist in higher complexity mixtures. *Here we plot the net interactions as a network, similar to main text figure 4, but focusing solely on the O-T interaction. Each point represents a different chemical mixture with the bottom row representing each individual chemical (designated by the first character of their name below the point) and every subsequent row above being a more complex mixture of these chemicals, finishing with a single point for the 8-chemical mixture. Nodes with significant interactions, which include the O-T combination, are larger, and coloured by antagonism (teal) or synergism (yellow). Dark edges (black) are drawn between nodes with significant interactions one row apart where the mixture below is a subset of the mixture above. Light edges (grey) are drawn between all possible O-T mixture subsets. For R. herbae and S. faeni, the O-T mixture is synergistic, and this interaction filters through to almost all other possible higher complexity O-T mixtures. C. gallinarum follows a similar pattern but with an antagonistic interaction. A. fischeri, A. humicola 2 and A. popoffii have much sparser networks, with a smaller subset of the higher complexity O-T mixtures retaining the 2-way antagonistic interaction. However, these networks are very similar, with the same nodes (chemical combinations) tending to retain the antagonistic interaction.*

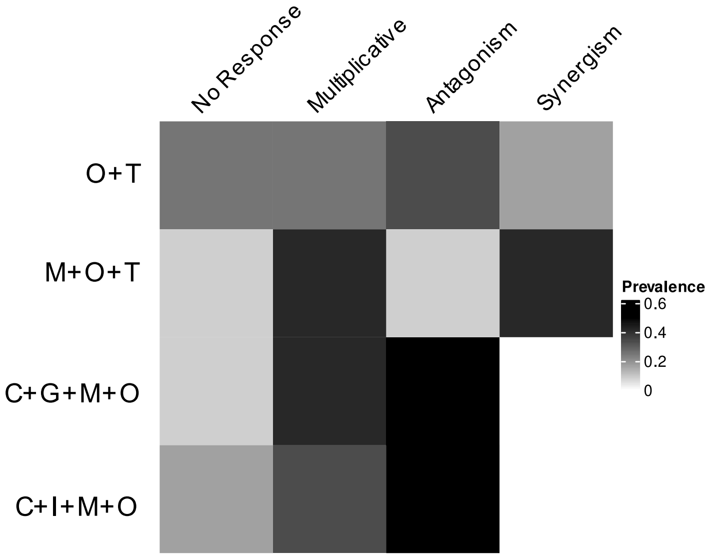

Figure S7 Most prevalent interactions across all strains. *Here we show the prevalence of response types in chemical combinations (C = chlorothalonil; G = glyphosate; I = imidacloprid; M = metaldehyde, O = oxytetracycline; T = tebuconazole) which show an interaction (antagonism or synergism) in at least half of the strains tested (≥6). The heatmap is coloured from white to black for the frequency of each response type across all strains (12), i.e., a prevalence of 0.5 means that that response type occurred in 6 strains. O+T is the only 2-way interaction with high prevalence among the strains tested. All of the more complex mixtures with prevalent interactions combine M and O.*

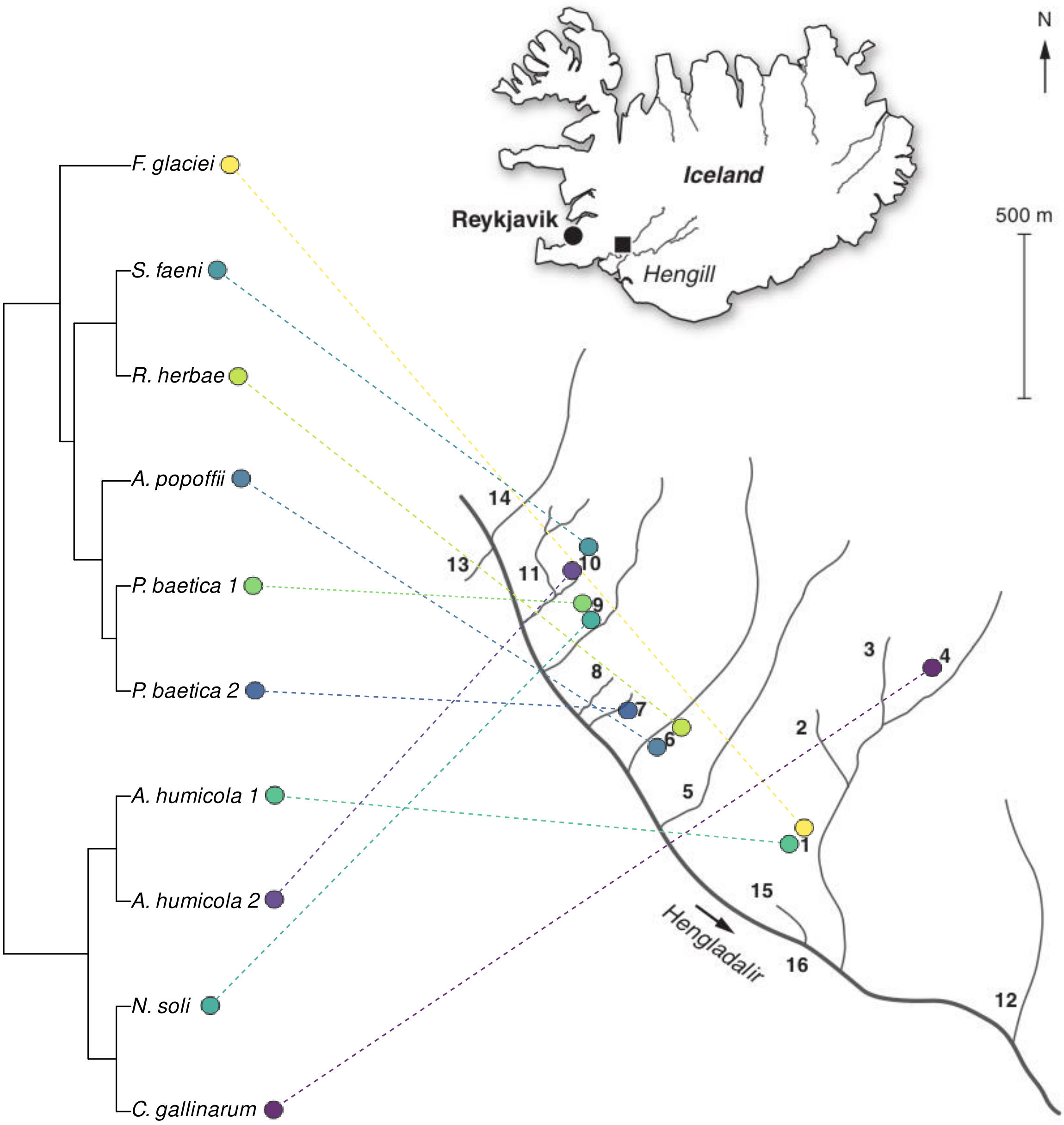

Figure S8 Iceland sampling sites for environmental bacteria. *Bacteria used were taken from a library of isolates extracted from stream sediments from the Hengill region of Iceland. Here we show the phylogeny of isolates (left) mapped on to the streams they were extracted from (right). We selected isolates with varied taxonomy from a range of the different streams sampled. Where isolates with the same species-level taxonomic ID were tested (A. humicola and P. baetica), these were isolated from different sites. Figure adapted from Woodward et al. (2010).*

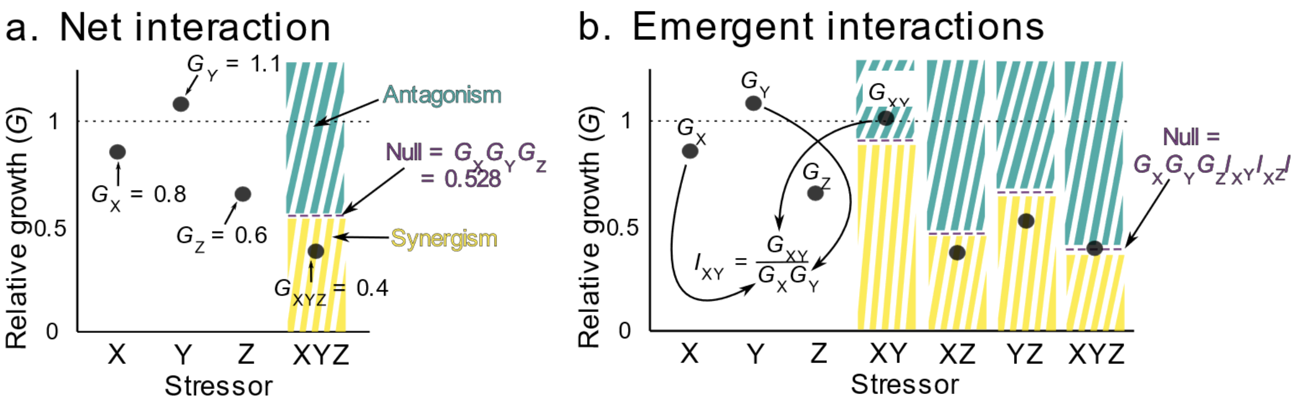

Figure S9 Detecting multi-stressor interactions on organismal growth under chemical mixtures. *We calculated the total growth (G) of single bacterial strain populations under different chemical stressors (XYZ), both individually and in mixtures, relative to growth under control conditions (i.e., no chemical stress, G = 1, indicated by dashed horizontal line. Values of G greater or less than 1 are increased or decreased growth, respectively).* ***a.*** *To calculate the net interaction of a chemical mixture we constructed a multiplicative null model based on the responses to each chemical individually (black points). We then calculated the interaction by comparing the growth response in the mixture (black point) to the null model expectations (purple dashed line) and asked if the response was stronger (synergistic; yellow area) or weaker (antagonistic; teal area) than predicted. In the example figure, the net interaction term (N_{XYZ}) is calculated as* $\frac{G_{XYZ}}{G_X G_Y G_Z}$. ***b.*** *To understand if the mixture contained an emergent interaction (i.e., a 3-way interaction in our example), we needed to account for all possible lower order interactions which may explain the combined response. We tested for 2-way interactions (I) by comparing the growth in mixtures to the null model expectations, and then incorporated those interactions into the null model when testing for a 3-way emergent interaction, i.e.,* $I_{XYZ} = \frac{G_{XYZ}}{G_X G_Y G_Z I_{XY} I_{XZ} I_{YZ}}$ *. We used this framework combined with a bootstrapping approach to test for significance of up to 8-way interactions in our experiments.*

Table S1: Results of phylogenetic signal tests. *All values calculated for Pagel's λ and Blomberg's K, corresponding to those shown in main text Figure 3 are given here, alongside their p-values from significance tests. Amoxicillin is highlighted in bold as the only chemical showing phylogenetic signal for either metric.*

Chemical	λ test statistic	λ p-value	K test statistic	K p-value
**Amoxicillin**	**0.824**	**0.037***	**0.885**	**0.029***
Chlorothalonil	>0.001	1	0.250	0.972
Diflufenican	0.555	0.241	0.656	0.417
Glyphosate	0.538	0.218	0.623	0.164
Imidacloprid	0.694	0.191	0.744	0.091
Metaldehyde	0.435	0.455	0.590	0.195
Oxytetracycline	>0.001	1	0.489	0.314
Tebuconazole	>0.001	1	0.385	0.651

* = $p < 0.05$

Table S2: List of bacterial strains used in this experiment. *Numbers following "Iceland" in the origin column refer to freshwater streams sampled at different locations in Iceland (see Supplementary Figure S8).*

Species ID	Phylum	Class	Origin
Arthrobacter humicola 1	Actinomycetota	Actinomycetia	Iceland 1
Arthrobacter humicola 2	Actinomycetota	Actinomycetia	Iceland 10
Carnobacterium gallinarum	Bacillota	Bacilli	Iceland 4
Neobacillus soli	Bacillota	Bacilli	Iceland 9
Flavobacterium glaciei	Bacteroidota	Flavobacteriia	Iceland 1
Rhizobium herbae	Pseudomonadota	Alphaproteobacteria	Iceland 6
Sphingomonas faeni	Pseudomonadota	Alphaproteobacteria	Iceland 10
Aeromonas popoffii	Pseudomonadota	Gammaproteobacteria	Iceland 6
Pseudomonas baetica 1	Pseudomonadota	Gammaproteobacteria	Iceland 9
Pseudomonas baetica 2	Pseudomonadota	Gammaproteobacteria	Iceland 7
Escherichia coli K12	Pseudomonadota	Gammaproteobacteria	Model strain
Aliivibrio fischeri	Pseudomonadota	Gammaproteobacteria	Model strain

Table S3: Chemical stressors used in our experiment. *Where possible, pesticide products with the chemical stressor as the active ingredient were used; manufacturers and product names are given. For the antibiotics and metaldehyde the purified forms of the chemicals were used; manufacturers and product numbers are given.*

Chemical stressor	Target effect	Manufacturer	Product
Glyphosate	Herbicide	Monsanto	Roundup Provantage
Diflufenican	Herbicide	ADAMA	Hurricane SC
Imidacloprid	Insecticide/Nematicide	Scotts	Intercept 70
Metaldehyde	Insecticide/Molluscicide	Sigma-Aldrich	63990
Tebuconazole	Fungicide	Provanto	Fungus Fighter
Chlorothalonil	Fungicide	Syngenta	Bravo 500
Amoxicillin	Antibiotic	TCI	A2099
Oxytetracycline	Antibiotic	LKT Labs	O9396

Table S4: Extended chemical details. *Here we provide effective concentrations for the chemicals used, alongside the target organisms for which those values were obtained. For the pesticides, these were obtained from the EPA EcoTox database (Olker 2022). For the antibiotics, minimum inhibitory concentrations (MIC) were obtained from the EUCAST clinical breakpoints database (EUCAST, 2023) for a representative bacterial species. Where possible, we report EC_{50}, where these were not available, we report alternative metrics. We also provide water solubility estimations and the references from which these were obtained. Inchem provides peer reviewed chemical safety information, collated by the World Health Organization. Pubchem is an open chemistry database hosted by the National Institutes of Health.*

Chemical	Concentration mg/L (endpoint, target)	Water solubility (at T°C)	Solubility Reference
Amoxicillin	0.125-1 (MIC, *Staphylococcus*)	3.43 g/L (25°C)	Pubchem[1]
Chlorothalonil	0.05-500 (mortality, fungi)	0.6-1.2 mg/L (20°C)	Inchem[2]
Diflufenican	0.001-0.008 (EC_{50}, algae)	1.04 mg/L (50°C)	Calculated here*
Glyphosate	0.003-1082 (EC_{50}, algae)	12 g/L (25°C)	Inchem[3]
Imidacloprid	0.0002-71 (EC_{50}, invertebrates)	0.6 g/L (20°C)	Inchem[4]
Metaldehyde	0.69-7.4 (EC_{50}, invertebrates)	0.2 g/L (17°C)	Inchem[5]
Oxytetracycline	0.125-1 (MIC, *Staphylococcus*)	0.3 g/L (25°C)	Pubchem[6]
Tebuconazole	8-10 (EC_{100}, fungi)	36 mg/L (20°C)	Pubchem[7]

1. https://pubchem.ncbi.nlm.nih.gov/compound/Amoxicillin#section=Solubility
2. https://www.inchem.org/documents/ehc/ehc/ehc183.htm
3. https://www.inchem.org/documents/icsc/icsc/eics0160.htm
4. https://www.inchem.org/documents/icsc/icsc/eics1501.htm
5. https://inchem.org/documents/pims/chemical/pim332.htm
6. https://pubchem.ncbi.nlm.nih.gov/compound/Oxytetracycline#section=Solubility
7. https://pubchem.ncbi.nlm.nih.gov/compound/Tebuconazole#section=Solubility
** see calculation below.*

Diflufenican water solubility calculation

The water solubility of diflufenican in weight per volume is not reported in the public Inchem or Pubchem databases. Xu *et. al.* (2020) report diflufenican water solubility expressed as mole fraction at 1.042×10^{-6}, which we convert to mg/L here. The mole fraction is the ratio of moles of solute to total moles of solute and solvent. We can calculate the molality (moles of solute per kg of solvent) from the mole fraction as:

$$\text{molality} = 1000 \times G/(A - GA)$$

where G is the mole fraction and A is the molar weight of the solvent in g/mol (18.02 for water). Therefore, molality = 5.78×10^{-5}. The concentration in g/L is given by:

$$concentration \ = \ molarity \ \times \ molar\ weight$$

where molarity is the moles of solute per litre of solvent. Given that 1kg water = 1L; molality = molarity. Therefore, diflufenican water solubility = 0.00104 g/L = 1.04 mg/L.

References

Olker, J. H., Elonen, C. M., Pilli, A., Anderson, A., Kinziger, B., Erickson, S., Skopinski, M., Pomplun, A., LaLone, C. A., Russom, C. L. & Hoff, D. (2022) The ECOTOXicology Knowledgebase: A Curated Database of Ecologically Relevant Toxicity Tests to Support

Environmental Research and Risk Assessment. *Environmental Toxicology and Chemistry, 41*(6), 1520-1539.

The European Committee on Antimicrobial Susceptibility Testing (EUCAST). Breakpoint tables for interpretation of MICs and zone diameters. Version 13.1, 2023. http://www.eucast.org

Xu, R., Huang, C. & Zhang, H. (2020). Diflufenican Dissolved in Different Aqueous Cosolvency Mixtures: Equilibrium Solubility Measurement and Thermodynamic Modeling. *J. Chem. Eng. Data 65*(11).
