# Supplementary material for: High-throughput characterization of bacterial responses to complex mixtures of chemical pollutants
Source: Nat Microbiol. 2024 Mar 18;9(4):938–48. doi: 10.1038/s41564-024-01626-9 (PMC10994839; doi:10.1038/s41564-024-01626-9)
Supplement: Supplementary file 2 — Reporting Summary [file 41564_2024_1626_MOESM2_ESM.pdf]

nature portfolio

Corresponding author(s): Thomas P. Smith

Last updated by author(s): Jan 26, 2024

Reporting Summary

Nature Portfolio wishes to improve the reproducibility of the work that we publish. This form provides structure for consistency and transparency in reporting. For further information on Nature Portfolio policies, see our Editorial Policies and the Editorial Policy Checklist.

Statistics

For all statistical analyses, confirm that the following items are present in the figure legend, table legend, main text, or Methods section.

n/a	Confirmed	
☐	☒	The exact sample size (*n*) for each experimental group/condition, given as a discrete number and unit of measurement
☐	☒	A statement on whether measurements were taken from distinct samples or whether the same sample was measured repeatedly
☐	☒	The statistical test(s) used AND whether they are one- or two-sided<br>*Only common tests should be described solely by name; describe more complex techniques in the Methods section.*
☒	☐	A description of all covariates tested
☐	☒	A description of any assumptions or corrections, such as tests of normality and adjustment for multiple comparisons
☐	☒	A full description of the statistical parameters including central tendency (e.g. means) or other basic estimates (e.g. regression coefficient) AND variation (e.g. standard deviation) or associated estimates of uncertainty (e.g. confidence intervals)
☐	☒	For null hypothesis testing, the test statistic (e.g. *F*, *t*, *r*) with confidence intervals, effect sizes, degrees of freedom and *P* value noted<br>*Give P values as exact values whenever suitable.*
☒	☐	For Bayesian analysis, information on the choice of priors and Markov chain Monte Carlo settings
☒	☐	For hierarchical and complex designs, identification of the appropriate level for tests and full reporting of outcomes
☒	☐	Estimates of effect sizes (e.g. Cohen's *d*, Pearson's *r*), indicating how they were calculated

Our web collection on statistics for biologists contains articles on many of the points above.

Software and code

Policy information about availability of computer code

Data collection	No software was used for data collection.
Data analysis	Data analysis packages:<br>MAFFT (v7.205); RAxML (v8.1.1); R (version 4.2.1); Vegan R-package (version 2.6-4); Phytools R-package (version 1.2-0).<br>All custom R code for our analyses are available from: https://github.com/smithtp/isolate-chem-mixtures/

For manuscripts utilizing custom algorithms or software that are central to the research but not yet described in published literature, software must be made available to editors and reviewers. We strongly encourage code deposition in a community repository (e.g. GitHub). See the Nature Portfolio guidelines for submitting code & software for further information.

Data

Policy information about availability of data

All manuscripts must include a data availability statement. This statement should provide the following information, where applicable:

- Accession codes, unique identifiers, or web links for publicly available datasets
- A description of any restrictions on data availability
- For clinical datasets or third party data, please ensure that the statement adheres to our policy

The minimum dataset necessary to interpret, verify and extend the research is available to download from figshare: https://doi.org/10.6084/m9.figshare.25054913.v1

All data is also available in our GitHub repository: https://github.com/smithtp/isolate-chem-mixtures/
16S sequences for the isolates used have been deposited in NCBI GenBank under accession codes PP204206:PP204215.
The EPA EcoTox database accessed for effective concentrations is available at: https://cfpub.epa.gov/ecotox/

Research involving human participants, their data, or biological material

Policy information about studies with human participants or human data. See also policy information about sex, gender (identity/presentation), and sexual orientation and race, ethnicity and racism.

Reporting on sex and gender	N/A
Reporting on race, ethnicity, or other socially relevant groupings	N/A
Population characteristics	N/A
Recruitment	N/A
Ethics oversight	N/A

Note that full information on the approval of the study protocol must also be provided in the manuscript.

Field-specific reporting

Please select the one below that is the best fit for your research. If you are not sure, read the appropriate sections before making your selection.

☒ Life sciences ☐ Behavioural & social sciences ☐ Ecological, evolutionary & environmental sciences

For a reference copy of the document with all sections, see nature.com/documents/nr-reporting-summary-flat.pdf

Life sciences study design

All studies must disclose on these points even when the disclosure is negative.

Sample size	Sample sizes were not statistically predetermined. he general standards of the field are to provide at least 3 independent biological replicates for each condition (see e.g. https://doi.org/10.1002/bies.202100108). We struck a balance between maximizing statistical power whilst still leveraging the high-throughput nature of our experiments, by performing 4 independent biological replicates for each condition.
Data exclusions	Growth curves in negative control wells were inspected by eye for evidence of contamination. Where contamination was observed, data from the plate was excluded. 2.5% of growth curves were excluded.
Replication	Experimental growth assays were performed in 4 independent biological replicates. Where data was excluded (see exclusions), at least 3 biological replicates from each condition were taken forward for analysis.
Randomization	Randomization was not relevant as we were applying a uniform set of treatments to each strain of bacteria.
Blinding	Blinding was not relevant as each strain of bacteria had the same set of treatments applied to it, and data was then collected continuously by a robotic plate reader.

Reporting for specific materials, systems and methods

We require information from authors about some types of materials, experimental systems and methods used in many studies. Here, indicate whether each material, system or method listed is relevant to your study. If you are not sure if a list item applies to your research, read the appropriate section before selecting a response.

Materials & experimental systems

n/a	Involved in the study	
☒	☐	Antibodies
☒	☐	Eukaryotic cell lines
☒	☐	Palaeontology and archaeology
☒	☐	Animals and other organisms
☒	☐	Clinical data
☒	☐	Dual use research of concern
☒	☐	Plants

Methods

n/a	Involved in the study	
☒	☐	ChIP-seq
☒	☐	Flow cytometry
☒	☐	MRI-based neuroimaging
